# Ovarian carcinomas with genetic and epigenetic BRCA1 loss have distinct molecular abnormalities

**DOI:** 10.1186/1471-2407-8-17

**Published:** 2008-01-22

**Authors:** Joshua Z Press, Alessandro De Luca, Niki Boyd, Sean Young, Armelle Troussard, Yolanda Ridge, Pardeep Kaurah, Steve E Kalloger, Katherine A Blood, Margaret Smith, Paul T Spellman, Yuker Wang, Dianne M Miller, Doug Horsman, Malek Faham, C Blake Gilks, Joe Gray, David G Huntsman

**Affiliations:** 1 Department of Obstetrics and Gynecology, University of British Columbia, Vancouver, British Columbia, Canada; 2 Department of Pathology and Laboratory Medicine, University of British Columbia, Vancouver, British Columbia, Canada; 3 Hereditary Cancer Program, British Columbia Cancer Agency, Vancouver, British Columbia, Canada; 4 Genetic Pathology Evaluation Centre of the Prostate Centre, Vancouver General Hospital, Vancouver, British Columbia, Canada; 5 Molecular Genetics, The Royal Melbourne Hospital, Parkville, Australia; 6 Life Sciences Division, Lawrence Berkeley National Laboratory, University of California, San Francisco, California, USA; 7 Affymetrix Inc. 7300 Shoreline Blvd South, San Francisco, California, USA; 8 Department of Gynecologic Oncology, University of British Columbia, Vancouver, British Columbia, Canada

## Abstract

**Background:**

Subclassification of ovarian carcinomas can be used to guide treatment and determine prognosis. Germline and somatic mutations, loss of heterozygosity (LOH), and epigenetic events such as promoter hypermethylation can lead to decreased expression of BRCA1/2 in ovarian cancers. The mechanism of BRCA1/2 loss is a potential method of subclassifying high grade serous carcinomas.

**Methods:**

A consecutive series of 49 ovarian cancers was assessed for mutations status of BRCA1 and BRCA2, LOH at the BRCA1 and BRCA2 loci, methylation of the BRCA1 promoter, BRCA1, BRCA2, PTEN, and PIK3CA transcript levels, PIK3CA gene copy number, and BRCA1, p21, p53, and WT-1 immunohistochemistry.

**Results:**

Eighteen (37%) of the ovarian carcinomas had germline or somatic BRCA1 mutations, or epigenetic loss of BRCA1. All of these tumours were high-grade serous or undifferentiated type. None of the endometrioid (n = 5), clear cell (n = 4), or low grade serous (n = 2) carcinomas showed loss of BRCA1, whereas 47% of the 38 high-grade serous or undifferentiated carcinomas had loss of BRCA1. It was possible to distinguish high grade serous carcinomas with BRCA1 mutations from those with epigenetic BRCA1 loss: tumours with BRCA1 mutations typically had decreased PTEN mRNA levels while those with epigenetic loss of BRCA1 had copy number gain of PIK3CA. Overexpression of p53 with loss of p21 expression occurred significantly more frequently in high grade serous carcinomas with epigenetic loss of BRCA1, compared to high grade serous tumors without loss of BRCA1.

**Conclusion:**

High grade serous carcinomas can be subclassified into three groups: BRCA1 loss (genetic), BRCA1 loss (epigenetic), and no BRCA1 loss. Tumors in these groups show distinct molecular alterations involving the PI3K/AKT and p53 pathways.

## Background

Ovarian cancer is the most fatal gynecological cancer in North American women and the fifth most common cause of cancer death. Epithelial ovarian carcinomas (EOC) are subclassified according to tumor cell type and grade. These different subtypes of ovarian cancer are associated with different molecular characteristics: high grade serous cancers typically contain TP53 mutations [1,2], low grade serous carcinomas often have RAS-RAF pathway activation and mutations in the KRAS and BRAF genes [3], low-grade endometrioid cancers are associated with mutations in the beta-catenin gene, CTNNB1 [4], and mucinous cancers frequently have mutations in KRAS [5]. Accurate subclassification of ovarian cancers is essential because different subtypes of ovarian cancer respond differently to treatment and have different prognoses [6].

The majority of ovarian cancers are of serous subtype [7]. In a study of 220 ovarian carcinomas, over half were categorized as serous and over 90% of these serous ovarian cancers were high grade (grade II or III) [8]. High grade serous ovarian cancers are associated with BRCA1 or BRCA2 mutations [9-11]. BRCA1, located at 17q21 [12-14], and BRCA2, located at 13q12-q13 [15,16] both encode tumor suppressors involved in repairing double-stranded DNA breaks and maintaining genomic stability [17-19] Germline BRCA1 or BRCA2 mutations are present in 10% to 15% of all EOCs [20,21]. Less commonly, EOCs contain somatic mutations in these genes [22]. In addition, aberrant expression of BRCA1 or BRCA2 may occur through loss of heterozygosity or, in the case of BRCA1, promoter hypermethylation [23-27]. Unlike breast cancer, where patients with germline mutations in BRCA1 or BRCA2 have cancers that are distinct from sporadic breast cancers on the basis of morphology or gene profiling [28-30], ovarian cancers with germline BRCA1 or BRCA2 mutations are indistinguishable from their sporadic counterparts [11,23,31-33]. High grade serous ovarian cancers that have functional BRCA1 or BRCA2 are currently not separable from high grade serous cancers that have loss of function of these proteins, based on routine histopathological examination. The challenge is to find methods to distinguish these subtypes of high grade serous cancers; this would stratify patients based on the underlying molecular events during oncogenesis, which is potentially highly relevant as these cancers may respond differently to treatment. This has recently been demonstrated in vitro: inhibitors of poly(ADP-ribose) polymerase (PARP1) were found to be able to target and kill cells deficient in either BRCA1 or BRCA2 [34,35]. PARP1 binds to single-stranded DNA breaks, attracting proteins to repair DNA. Inhibition of PARP1 allows these single stranded breaks to progress to double stranded breaks through the resulting collapse of replication forks [34,36]. The preferred double-strand break repair mechanism is homologous recombination which relies on the activity of BRCA1 and BRCA2. Thus, cells with loss of BRCA1 or BRCA2 function that are treated with PARP1 inhibitors are unable to repair DNA breaks, which leads to crisis and cell death.

We collected and analysed 49 consecutive ovarian tumor samples from consenting women diagnosed with invasive, non-mucinous EOC who were undergoing debulking surgery. We pathologically characterized these samples and analysed them for BRCA1 and BRCA2 mutations, loss of heterozygosity at both loci, mRNA levels of BRCA1 and BRCA2, expression of BRCA1, and BRCA1 promoter hypermethylation. In light of previous studies showing that promoter hypermethylation of BRCA2 is rarely if ever encountered in ovarian carcinoma [37-39], we did not undertake similar studies on the BRCA2 promoter. We then attempted to determine whether molecular profiles could be used to distinguish high grade serous cancers with BRCA1 mutations (germline or somatic), from high grade serous cancers with epigenetic loss of BRCA1 through promoter hypermethylation, and high grade serous cancers without BRCA1 loss. We focused on the PI3K/AKT and p53 pathways which play important roles in ovarian cancer [40-43].

## Methods

### Recruitment and tumor samples

Between January 2004 and September 2005 all women undergoing primary debulking surgery for ovarian carcinoma were invited to participate in this study at the Vancouver General Hospital and British Columbia Cancer Agency in Vancouver, Canada. Women with mucinous and borderline tumors, and women who had received pre-operative chemotherapy were excluded. Pathology was reviewed by a single pathologist (CBG). Serous tumors were classified as low or high-grade as described previously [44]; all undifferentiated and clear cell carcinomas were considered high-grade. Endometrioid carcinomas were graded as grade 1, 2, or 3 according to the Silverberg grading system [45]. Ethical approval was obtained from the University of British Columbia Ethics Board (#H02-61375 and #H03-70606).

### DNA and RNA extraction

Cancer tissue was split with part stored at -80 degrees and the facing tissue fixed in formalin and placed in paraffin blocks. H&E sections were reviewed to ensure samples consisted of ≥ 70% tumor cells. DNA was extracted using the Puregene DNA Purification Kit (Gentra Systems, Inc, Wicklow, Ireland) according to manufacturer's instructions from whole blood (germline analysis) or tumor samples (somatic analysis). RNA was isolated with Trizol (Invitrogen, Carlsbad, CA) according to standard protocols.

### Loss of heterozygosity analysis

Somatic loss of BRCA1/BRCA2 in tumor tissue was assessed for LOH using microsatellite markers for BRCA1 (D17S855 (60°C), D17S1185 (58°C), D17S1323 (56°C), and D17S1325 (56°C)) [46], and BRCA2 (D13S260 (60°C), D13S171 (50°C), D13S267 (53°C), D13S217 (55°C)) [9]. PCR products were electrophoresed in an ABI Prism 3100 Genetic Analyzer (Applied Biosystems, Foster City, CA), and analyzed with Genescan v3.1 software (Applied Biosystems, Foster City, CA). LOH was defined as a complete or partial (≤ 50%) signal reduction of one allele in at least one marker. Microsatellite instability (MSI) was defined as the presence of novel alleles in the tumor DNA that were not present in normal DNA in at least one marker [47].

### dHPLC mutation screening and mutation analysis

Screening for BRCA1/BRCA2 mutations was performed using denaturing high performance liquid chromatography (dHPLC). Tumor DNA was mixed in a 3:1 ratio with corresponding germline DNA for all tumors shown to possess LOH to ensure that LOH did not mask somatic mutations [48]. For example, with intratumoral LOH, and mutation of the remaining allele, dHPLC screening would give a false negative result. If the mutation is a germline mutation it will be picked up on dHPLC screening of germline DNA, however, a somatic mutation would be missed in both tumor and germline DNA without DNA mixing. PCR primers and conditions were developed by the Royal Melbourne Hospital (Australia) and are available on request. PCR primers were used to amplify each exon of BRCA1 (24 exons) and BRCA2 (26 exons). All exons with abnormal dHPLC profiles were PCR amplified and bi-directionally sequenced to identify mutations using ABI BigDye terminator v3.1 cycle sequencing kit (Applied Biosytems, Foster City, CA) and an ABI Prism 3100 Genetic Analyzer (Applied Biosystems, Foster City, CA).

### Multiplex Ligation-dependent Probe Amplification (MLPA) screening

For the identification of germline BRCA1 single and multiple exon deletions or duplications, multiplex ligation-dependent probe amplification analysis (MLPA) kits SALSA P002 BRCA1 and SALSA P087 BRCA1 (MRC Holland, Amsterdam, NL) were used according to manufacturer directions. A reduction or increase in RPA values to <0.7 or >1.3 was considered an indication of a deletion or a duplication, respectively [49].

### BRCA1 and FANCF promoter hypermethylation analysis

The BRCA1 methylation status of each tumor was assessed using a technique similar to the MethyLight assay described previously [50]. Briefly, 500 ng of sample DNA was subjected to sodium bisulfite modification using the EZ DNA Methylation-Gold Kit, as recommended by the manufacturer (Zymo Research, Orange, CA). After bisulfite treatment, DNA was amplified using real-time PCR with oligonucleotide primers complementary to a region of the MYOD1 promoter that did not contain any CpG dinucleotides but did contain non-CpG cytosines to ascertain the amount of converted input templates in each sample. Hypermethylation of the BRCA1 promoter was then examined by real-time PCR amplification of bisulfite-modified DNA using oligonucleotide primers specific for a fully methylated bisulfite-converted portion of BRCA1 promoter such that only CpG islands that were methylated at every CpG dinucleotide interrogated by the primers and probes would be amplified and generate fluorescent signal. The sequences of the primers used to amplify and detect methylated BRCA1 promoter region were 5'-TAGAGTTTCGAGAGACGTTTGGTTT-3' (forward primer) and 5'-CGCTTTTCCGTTACCACGA-3' (reverse primer). The primers for MYOD1 were 5'-CCA ACTCCA AATCCCCTC TCTAT-3' (forward primer) and 5'-TGATTAATTTAGATTGGGTTTAGAGAAGGA-3' (reverse primer). The amount of methylated DNA (PMR, percentage of methylated reference) [51] at the BRCA1 locus was calculated by dividing the BRCA1: MYOD1 ratio of a sample by the BRCA1: MYOD1 ratio of CpG methylated Jurkat genomic DNA (New England Biolabs, Ipswich, MA) and multiplying by 100. Reactions using CpG methylated Jurkat genomic DNA were used to normalize for any difference in amplification efficiencies between BRCA1 and MYOD1. The PMR serves as an index of the percentage of bisulfite converted input copies of DNA that are fully methylated at the primer hybridization sites. The PMR values obtained by MethyLight were dichotomized at 4 PMR for statistical purposes as described previously [51]. Samples containing 4 PMR or higher were designated as methylated, whereas samples containing less than 4 PMR were designated as unmethylated. It is important to note, however, that the PMR may be >1 if copies of MYOD1 are deleted relative to the gene of interest, or copies of the gene of interest are gained relative to MYOD1 in any given sample. PCR experiments were carried out in a volume of 10 μL with 384-well plates and an Applied Biosystems 7900 HT Sequence Detector (Applied Biosystems, Foster City, CA). The fluorescence signal of the quantitative methylation-specific PCR was generated by SYBR Green I. Samples (10 ng bisulfite-treated DNA) were run in triplicate containing 5 μL SYBR Green Master Mix (Applied Biosystems, Foster City, CA) and 5 pmol of each forward and reverse primer. Bisulfite-converted CpG methylated Jurkat Genomic DNA (New England Biolabs, Ipswich, MA) served as a positive control and was used to generate a standard curve to quantify the amount of fully methylated promoters in each reaction. PCR amplification was done by means of the following procedure: 95°C for 15 minutes, followed by 40 cycles at 95°C for 15 seconds, 62°C for 1 minute. A subsequent dissociation curve analysis checked the specificity of products. FANCF promoter hypermethylation was assessed using a HpaII digest assay and methylation-specific PCR protocol previously reported by Taniguchi et al [52].

### Real-time Q-RT-PCR

Extracted RNA was treated with DNAse I (Invitrogen, Carlsbad, CA) prior to creating cDNA using random hexamer priming and MMLV reverse transcriptase (Invitrogen, Carlsbad, CA). Applied Biosystems Taqman primer/probe kits (Hs00173233_m1 (BRCA1), Hs00609060_m1 (BRCA2), Hs01920652_s1 (PTEN), Hs00907966_m1(PIK3CA)) were used to quantify mRNA expression levels using real-time qRT-PCR [37] and an ABI Prism 7900 HT Sequence Detection System (Applied Biosystems, Foster City, CA). Relative gene expression quantification was calculated according to the comparative Ct method using human 18S ribosomal RNA (Applied Biosystems, Foster City, CA) and commercial RNA controls (Stratagene, La Jolla, CA). Relative quantification was determined as follows: 2^-(ΔCt sample-ΔCt calibrator)^. Ratios (tumor relative gene expression:average of all tumors) less than 0.7 or greater that 1.3 for were scored as decreased or increased mRNA expression, respectively.

### Immunohistochemistry

The BRCA1 antibody Ab-1 (Oncogene, 1:50 dilution) was used and antigen retrieval was performed in 1× EDTA buffer (pH 8.0) by microwaving for 2 minutes, and then boiling in a waterbath for 30 minutes. Endogenous peroxide activity was blocked with 3% hydrogen peroxide and then sections were incubated with 2.5% normal horse blocking serum. Following incubation with the primary antibody, the Vector Laboratories (Burlingame, CA) ImmPRESS kit was used according to the manufacturer's recommendations to visualize antibody complexes. Nuclear staining was assessed by CBG, who was blinded to all other BRCA analysis. Tumors were considered BRCA1 positive if greater than 1% of tumor nuclei showed staining. IHC was also performed with the following panel of previously validated antibodies using a Ventana (Tucson, AZ) automated immunostainer: p21 (Neomarkers, Fremont, CA, clone DCS-60.2, 1:100 dilution), p53 (Dako, Carpinteria, CA, clone DO-7, 1:400 dilution), and WT-1 (Dako, Carpinteria, CA, clone 6F-H2, 1:50 dilution). BRCA1 IHC was done on whole sections, while other IHC markers were assessed using sections from a tissue microarray constructed with two 0.6 mm cores per case.

### Molecular Inversion Probe (MIP) Copy Number

The MIP copy number assay was done as described previously [53] with some modifications. Specifically, the current protocol is a modification of the Targeted Genotyping protocol commercialized by Affymetrix [54]. Test DNA samples were diluted to16 ng/μl. Molecular inversion probes were annealed to DNA by mixing 4.7 μl of DNA (75 ng total), 0.75 μl of Buffer A, 1.1 μl of the 53 K molecular inversion probe pool (200 amol/μl/probe) and 0.045 μl of Enzyme A in a 384-well plate on ice. The reaction was incubated for 4 min at 20°C, 5 min at 95°C, then overnight at 58°C. Following annealing, 13 μl of Buffer A and 1.25 μl of Gap Fill Enzyme mix were added to each reaction and 9 μl of reaction volume was transferred to each of two wells in a 96-well plate. Molecular inversion probes were circularized with 4 μl of dNTP mix at 58°C for 10 min. Linear probes and genomic DNA were eliminated by addition of 4 μl of Exo Mix and incubation at 37°C for 15 min, followed by universal primer amplification for 18 cycles (20 sec at 95°C, 40 sec at 64°C, and 10 sec at 72°C). For labelling reactions, the product was further amplified for 10 cycles using labelled primers, then subjected to cleavage by HY Digest Mix at 37°C for 2 hours. The cleaved MIP products were mixed with Hybridization Cocktail, denatured, and hybridized to 70 K Universal Taq arrays at 39°C for 16 h (two arrays per sample). The overnight hybridized arrays were washed on a GeneChip^® ^Fluidics Station FS450 and stained by SAPE at 5 ng/ml (Invitrogen).

Copy number estimation was obtained from the hybridization signals as previously described [55], with the following modifications: the color-separation step was omitted as the single color readout on two arrays prevented the spectral overlap that occurs with multi-color readouts, and Langmuir correction was performed instead of linear calibration of allele signals [56]. Copy numbers over 3.0 were considered amplification events and copy numbers below 1.5 were considered deletion events.

### Data analysis

Epigenetic BRCA1 loss was defined as having promoter hypermethylation accompanied by either low relative BRCA1 mRNA expression, negative BRCA1 IHC, or both low BRCA1 mRNA and negative BRCA1 IHC. Tumors without promoter hypermethylation and with positive BRCA1 IHC were not considered to have BRCA1 loss. Tumors with negative BRCA1 IHC without promoter hypermethylation were considered equivocal for BRCA1 loss. A chi-squared test or Fisher exact test was used to analyze categorical variables (MIP copy number, IHC) and a student's t-test was used to analyze continuous variables (RNA expression).

## Results

**Figure 1:**
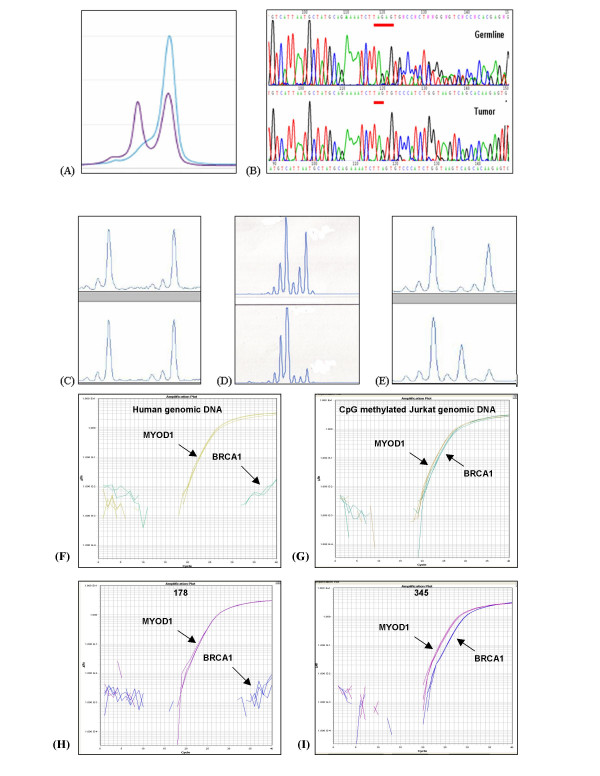
Assessment of BRCA1 loss (A) Mutation screening showing the abnormal denaturing high performance liquid chromatography profile corresponding to the 1351delAT mutation in tumor 223. The single blue line represents the electropherogram from a normal control, while the purple line represents the abnormal profile formed by the mutated exon 11c in tumor 223. (B) Direct DNA sequencing demonstrating the 185delAG mutation in tumor 283. Only the mutant allele is seen in the tumor because LOH is present. (C-E) Loss of heterozygosity (LOH) analysis using BRCA1-associated microsatellite markers visualized on an ABI Prism 3100 Genetic Analyzer, where LOH is defined as >50% decrease in area under the curve when germline DNA (upper tracing) and tumor DNA (lower tracing) are compared. (C) The lack of LOH in tumor 240 demonstrated using microsatellite marker D17S1185, (D) LOH in tumor 283 demonstrated using microsatellite marker D17S855. (E) Microsatellite instability demonstrated in tumor 156 using microsatellite marker D17S1185. (F, G, H, and I) Methylation analysis of BRCA1 gene using fluorescence-based, quantitative, real-time PCR (TaqMan) using SYBR Green 1 as detection method. Two sets of primers, designed specifically for bisulfite converted DNA, were used: a methylated set for the BRCA1 gene and a reference set (MYOD1) to control for input DNA. Specificity of the reactions for methylated DNA were confirmed separately using human genomic DNA (unmethyated; F) and CpG methylated Jurkat genomic DNA (methylated; G), respectively. H and I show representative examples of results from assessment of BRCA1 loss through promoter hypermethyation. Tumor 178 shows only unmethylated BRCA1 promoter, while tumor 345 shows evidence of BRCA1 promoter hypermethylation.

Representative results of analysis for BRCA1 mutations, BRCA1 loss of heterozygosity, and BRCA1 promoter hypermethylation are shown in Figure 1.

**Figure 2:**
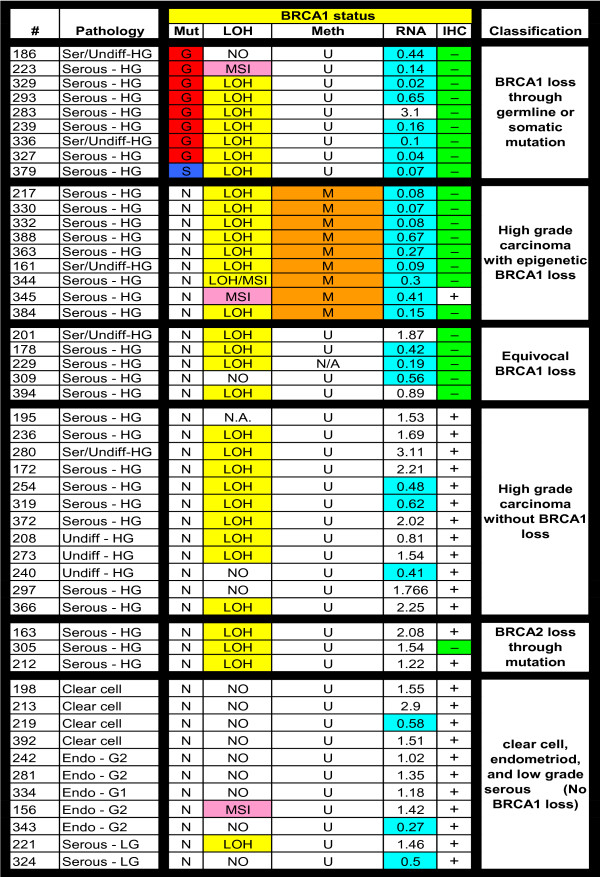
Summary of BRCA1 abnormalities and associated features: Pathology refers to the tumor histopathology. Serous or Ser = serous carcinoma; Undiff = undifferentiated carcinoma; HG = high-grade; LG = low-grade; Clear cell = clear cell carcinoma; Endo = endometrioid carcinoma; G1 = grade 1; G2 = grade 2; G3 = grade 3. BRCA1 Status: Mut = mutation; G = germline; S = somatic; N = no mutations. LOH = loss of heterozygosity where LOH indicates that loss of heterozygosity is present, NO indicates that loss of heterozygosity is not present, and MSI indicates that microsatellite instability is present in the tumor. Meth refers to BRCA1 promoter hypermethylation. Tumors containing ≥ 4% fully methylated molecules are designated as methylated (M) and are highlighted in orange, whereas tumors containing < 4% are designated as unmethylated (U). RNA refers to relative RNA expression compared to the average RNA expression in all samples, where the average RNA expression = 1.0. Tumors with relative RNA expression <0.7 are highlighted in aqua as showing BRCA1/BRCA2 loss. IHC refers to BRCA1 immunohistochemistry; (+) indicates tumors with > 1% of nuclei stained positive for BRCA1, (-) indicates tumors with <1% of nuclei positive. N/A indicates that the data is not available for technical reasons.

BRCA1 findings for all tumors are presented as Figure 2. Based on these results, ovarian cancers were divided into six groups: (1) BRCA1 mutations, (2) BRCA1 epigenetic loss, (3) equivocal for BRCA1 loss, (4) high grade serous/undifferentiated cancers without BRCA1 loss, (5) BRCA2 mutations, and (6) clear cell, endometrioid, and low grade serous cancers.

Nine of the samples (18%) carried BRCA1 mutations (eight germline, one somatic) and three samples (6%) had BRCA2 mutations (two germline, one somatic). No intragenic deletions in BRCA1 were detected by MLPA analysis. Nine samples (18%) were found to have loss of BRCA1 due to epigenetic events; these samples all had hypermethylation of the BRCA1 promoter accompanied by decreased BRCA1 mRNA levels (relative qRT-PCR expression <0.7) and/or lack (less than 1% of tumour nuclei) of BRCA1 immunohistochemical staining. Five samples (10%), which did not carry BRCA1 mutations, were all unmethylated at the BRCA1 promoter; these were classified as "equivocal for BRCA1 loss" because they fulfilled two of the following criteria: loss of heterozygosity (LOH) at the BRCA1 locus, decreased BRCA1 mRNA levels, or lack of BRCA1 immunohistochemical staining. All samples (n = 26) with BRCA1 or BRCA2 mutations, epigenetic loss of BRCA1, or classified as equivocal for BRCA1 loss were of high grade serous/undifferentiated type. Twelve tumors (24%) of high grade serous/undifferentiated type did not have BRCA1 mutations or epigenetic loss, or BRCA2 mutations. Thus, considering only the 38 high grade serous/undifferentiated tumours in our study, 18 (18/38, 47%) had loss of BRCA1 either through genetic or epigenetic events, three (3/38, 8%) carried germline or somatic BRCA2 mutations, 12 (12/38, 32%) had preservation of BRCA1 expression and no mutations in BRCA1 or BRCA2, and the remaining five tumors (5/38, 13%) were equivocal for BRCA1 loss and did not have BRCA2 mutations. FANCF promoter hypermethylation was not observed in any of these cases.

The remaining 11 tumour samples (22%) (i.e. those cases other that the high grade serous/undifferentiated carcinomas) were either clear cell (4), endometrioid (5), or low grade serous type (2). These samples were all negative for BRCA1 or BRCA2 mutations, negative for BRCA1 promoter hypermethylation, and positive for BRCA1 expression as determined by immunohistochemistry.

**Figure 3:**
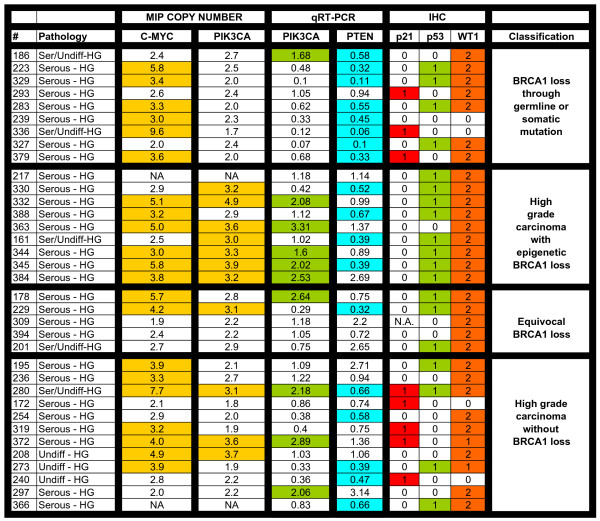
Summary of analysis of high grade (HG) serous/undifferentiated ovarian tumors: MIP copy number results are shown for c-myc and PIK3CA loci. MIP copy number values over 3.0 are highlighted and correspond to amplification. Relative mRNA levels for PIK3CA and PTEN were assessed using qRT-PCR; levels over 1.3 (highlighted in green) are considered elevated and levels below 0.7 (highlighted in aqua) indicate decreased transcript levels. Associated immunohistochemical markers p21, p53, and WT-1 refer to immunohistochemical staining results. Scoring of immunostaining was done as follows: p21: 0 = <5% nuclei positive and 1 = >5% of nuclei positive. p53: 0 = <50% nuclei positive and 1 = >50% of nuclei positive. WT1: 0 = <5% nuclei positive, 1 = 5–50% nuclei positive, and 2 = >50% nuclei positive. N/A indicates that the data is not available for technical reasons.

**Figure 4:**
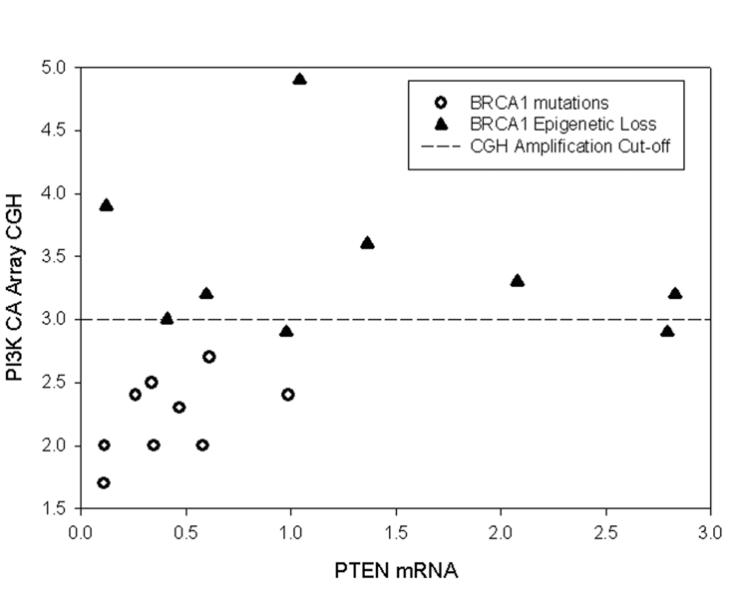
Correlation between decreased PTEN mRNA levels and amplification at the PIK3CA locus: Relative PTEN mRNA levels as determined by qRT-PCR are plotted along the X-axis and PIK3CA MIP copy number results are plotted along the Y-axis for high grade serous ovarian tumors with BRCA1 mutations (open circles) and high grade serous ovarian tumors with epigenetic loss of BRCA1 (filled triangles). MIP copy number values over 3.0 indicate amplification.

We further analyzed the 35 high grade serous/undifferentiated tumour samples that did not contain BRCA2 mutations using a combination of MIP copy number, qRT-PCR, and immunohistochemistry in order to determine whether these different groups could be classified according to specific molecular features other than BRCA1 or BRCA2 abnormalities (Figure 3). The number of tumors with BRCA2 mutations (n = 3) was considered too small for meaningful further analysis of this subset and was therefore excluded. Thirty-one of these tumours were positive for WT1 expression by immunohistochemistry, a marker of serous cell type in EOC, thus confirming our histopathological subclassification [57]; all clear cell and endometrioid cancers in this study were negative for WT1 expression (data not shown). Currently, the group of high grade serous/undifferentiated carcinoma is indivisible based on morphology or routinely used diagnostic molecular markers. We specifically focused on the PI3K/AKT and p53 pathways which are known to be important in EOC. We found that those tumours with BRCA1 loss through genetic events differed according to several parameters from tumours with loss of BRCA1 due to epigenetic events. Most striking were differences in PIK3CA copy number as determined by the MIP copy number assay. While none of the BRCA1 mutation positive cases demonstrated an increased PIK3CA copy number almost all (7/8) of the samples with epigenetic loss of BRCA1 had increased copy number at the PIK3CA locus. The PIK3CA copy number increases were low level (mean amplification ratio 2.7, range 1.7–4.9), and in all but one case amplification of PIK3CA was associated with amplification of the entire chromosomal arm. PIK3CA mRNA levels were assessed using qRT-PCR and relative mRNA levels were found to correlate with copy number ratios (p = 0.02). Specificity of MIP copy number data was verified by assessing c-myc amplification; while PIK3CA copy number appeared to be selectively increased in specific subgroups, amplification at the c-myc locus was observed at similar frequencies in all subgroups. Interestingly, decreased PTEN mRNA levels observed in cancers with BRCA1 mutations and increased PI3KCA copy number in cancers with epigenetic loss of BRCA1 were almost mutually exclusive (Figure 4). These events represent two separate mechanisms of activation of the canonical PI3K/AKT pathway.

**Figure 5:**
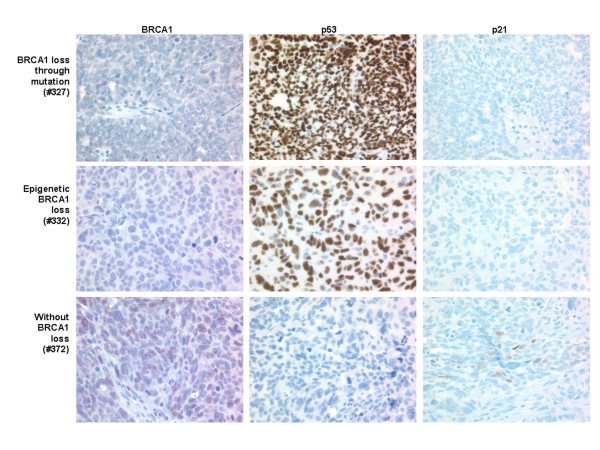
Immunohistochemistry results: Representative immunohistochemistry results for high grade serous ovarian tumors with BRCA1 mutations (tumor #327, top row), with epigenetic loss of BRCA1 (tumor #332, middle row), and without loss of BRCA1 (tumor #372, bottom row). Immunohistochemical staining is shown for BRCA1 (left column), p53 (middle column) and p21 (right column).

We further compared those tumors with either genetic or epigenetic loss of BRCA1 with high grade serous cancers without evidence of BRCA1 loss (Table 1). Representative immunohistochemical images for BRCA1, p53, and p21 from all three of these groups are shown in Figure 5. MIP copy number analysis of the nine tumours that had BRCA1 mutations showed that there was no loss or gain at the region of chromosome 3 that contains the gene for PIK3CA and only four (44%) samples stained positively for p53. By contrast, of the samples that had loss of BRCA1 due to epigenetic events, seven of eight samples (88%) had an increased PIK3CA copy number, and eight of nine samples (89%) stained positively for p53. In addition, high grade serous cancers without BRCA1 loss had low frequencies of positive p53 staining (4/12, 33%) and increased PIK3CA copy number (3/12, 25%), similar to cancers with BRCA1 mutations but distinct from cancers with epigenetic loss of BRCA1. In two cases (#366 and #217) we were unable to obtain MIP copy number data. It was noted that positive p53 staining was most often accompanied by negative p21 staining. The expression of p21 is increased in response to p53. High level overexpression of p53 correlates with p53 mutations and loss of function and would be anticipated to be associated with decreased p21 expression, as we observed. This p53+/p21- immunophenotype was significantly more common in tumors with BRCA1 epigenetic loss than in tumors without BRCA1 loss (Table 1).

**Table 1:** Comparison of PIK3 CA amplification or p53/p21 protein expression in High Grade Serous Ovarian Tumours

**Group**	**PIK3CA amplification (MIP copy number)**	**p53/p21 IHC**		
		**+/-**	**+/+, -/-**	**-/+**
**BRCA1 loss through mutation**	0/9^*+^	4/9	2/9	3/9
**BRCA1 epigenetic loss**	7/8^*+^	8/9**	1/9	0/9
**No BRCA1 loss**	3/11^+^	3/12**	5/12	4/12

* p = 0.02 for pairwise comparison based on Bonferroni-Holmes corrected Fisher exact test

** p = 0.03 for pairwise comparison based on Bonferroni-Holmes corrected Fisher exact test

+ p = 0.001 for threeway comparison based on Bonferroni-Holmes corrected Fisher exact test

## Discussion

The sub-classification of ovarian carcinomas, which is based on histopathological subtype and grade, is unable to adequately predict prognosis or response to treatment. Administration of adjuvant platinum/taxane chemotherapy is the standard treatment for ovarian carcinoma following debulking surgery [58]. Approximately 70% of advanced-stage ovarian carcinomas, however, will recur with development, ultimately of platinum-resistant disease [59]. A comparison of clinical outcomes between ovarian cancer patients with BRCA1 promoter hypermethylation to patients with BRCA1 mutations and wild-type BRCA1 genes demonstrated that patients with BRCA1 promoter hypermethylation had significantly shorter survival times compared to the other two groups [60]. Ovarian carcinomas in patients with BRCA1, in contrast are associated with a favourable prognosis [23,61]. This data suggests that different abnormalities in BRCA1 could be associated with different clinical outcomes and possibly distinct alterations in other underlying molecular abnormalities. For example, the colon cancers from patients with inherited mutations in DNA mismatch repair genes differ from sporadic cancers with microsatellite instability due to hypermethylation of the MLH1 promoters [62], with respect to age of onset of disease, pathology, and molecular alterations [63].

We evaluated 49 ovarian carcinomas and categorized them according to pathology and BRCA1 and BRCA2 status. We further evaluated 35 high grade serous/undifferentiated tumours that we divided into four groups based on BRCA1 mutation status, expression, and promoter hypermethylation. We observed increased positive p53 immunohistochemical staining, which correlated with negative p21 immunostaining, in cancers with epigenetic BRCA1 loss, compared to cancers with BRCA1 mutations and high grade serous/undifferentiated cancers without BRCA1 loss. p53 is a tumor suppressor that is involved in the progression of many cancers and is the most commonly mutated gene in ovarian carcinomas [37]. Typically, mutations in p53 result in accumulation of p53 in the nucleus and the majority of cases with abundant p53 detectable by immunohistochemistry are p53 mutant [64,65]. The p53 protein is an important mediator of apoptosis resulting from DNA damage, stress, or chemotherapy [66]. p21 is a downstream effector of the cell cycle arrest function of p53 and is upregulated at the transcriptional level by wildtype but not mutated p53 [67]. At present, p53 mutation status or expression is not used to guide clinical decisions [68]. We have observed that p53 overexpression correlates, as expected, with loss of p21 expression. Furthermore the phenotype of p53 overexpression with loss of p21 is significantly more common is high grade serous/undifferentiated tumors with epigenetic loss of BRCA1 compared to high grade serous/undifferentiated tumors without loss of BRCA1.

In addition, we found that ovarian carcinomas with loss of BRCA1 through genetic events and those with BRCA1 loss through epigenetic events both have activation of the PI3K/AKT pathway, though the mechanism of activation is different. PI3K phosphorylates phosphatidylinositol lipids in response to activation by receptor tyrosine kinases [69]. Its activity has been linked to proliferation, differentiation, cell adhesion, apoptosis, tumorigenesis, and angiogenesis [70]. PTEN is a phosphatase whose activity counters PI3K. The serine/threonine kinase AKT is a downstream target of PI3K [71] and the activity of one of its isoforms, AKT1, is elevated in ovarian carcinomas [72]. Both decreased PTEN levels and amplification of PIK3CA lead to increased phosphorylation of AKT and it has been previously shown that ovarian cancers often have alterations in PI3K and PTEN [73-75]. This is the first study, however, to report that decreased PTEN expression levels are associated with ovarian carcinomas carrying BRCA1 mutations while increased PI3KCA copy number is associated with ovarian carcinomas with epigenetic loss of BRCA1. It has been previously demonstrated in breast cancer and glioblastoma that PIK3CA mutations and PTEN loss are virtually mutually exclusive, suggesting that abrogation of just one of these proteins is sufficient for tumorigenesis [76,77]. We observe a similar result in our ovarian serous cancer samples; it is likely that deregulation of this pathway, while critical for tumorigenesis, can be accomplished through alteration of a single key molecule at which point selective pressure is relieved for altering other proteins involved in this signalling pathway. Our findings in serous ovarian carcinoma, and the previous results from studies of breast cancer and glioblastoma, are in contrast to ovarian carcinoma of endometrioid type [78], and endometrial cancer [79,80], where PTEN mutations and PIK3CA mutations frequently co-exist. This is yet another example of molecular events during the genesis of ovarian cancer that show tumor cell type specificity, and reinforces the need to consider cell type differences in studies of ovarian cancer pathogenesis.

We would expect that cancers with activation of the PI3K/AKT pathway may not respond well to common chemotherapy, as overexpression of activated AKT decreases apoptosis induced by paclitaxel in ovarian cancer cells [81] and introduction of the catalytic subunit of PI3K into ovarian cancer cells causes resistance to paclitaxel [82]. In addition, the PI3K inhibitor, LY294002, has been shown to decrease growth of ovarian carcinoma and ascites formation in mouse xenograft models of ovarian carcinoma [83]. As therapies continue to be developed that target the PI3K/AKT pathway, it will be essential to understand the molecular alterations that are affecting this pathway in different types of ovarian carcinomas.

The need for meaningful sub-classification of ovarian carcinoma is critical for improving the treatment and prognosis of patients. Though sub-classification may be done based on BRCA1 genetic testing, this cannot be done in a timely fashion such that it could be used to guide therapy of patients newly diagnosed with ovarian cancer. This is extremely important as patients must embark on therapy shortly after diagnosis. In addition, as new therapeutics are developed, rapid identification of appropriate patients will be necessary for clinical trials. Our results demonstrate that it may be possible to categorize patients based on rapid molecular tests to identify patients who are likely to harbour BRCA1 mutations. Negative BRCA1 immunohistochemical staining, decreased BRCA1 mRNA, lack of PI3K amplification, and absence of BRCA1 promoter hypermethylation is an example of a molecular profile that could be used to identify these patients. This would also allow more cost effective and efficient mutation screening in patients presenting with ovarian carcinoma.

## Conclusion

This is the first study to comprehensively examine data from detailed analysis of BRCA1 and BRCA2 abnormalities in ovarian cancer. Results presented here demonstrate that high grade serous/undifferentiated carcinomas can be subclassified based on the underlying BRCA abnormalities. Such clinically relevant subclassification is critical for developing specific treatements for ovarian cancer patients which will lead to improved prognosis and management of disease.

## Competing interests

The author(s) declare that they have no competing interests.

## Authors' contributions

JZP participated in the study design and coordination, patient recruitment, sample collection and handling, molecular analysis, immunohistochemical analysis, and drafting of the manuscript. AD participated in the tumor analysis, molecular analysis, and drafting of the manuscript. NB contributed to drafting of the manuscript. SY participated in the BRCA1/2 germline mutational analysis. AT participated in drafting of the manuscript. YR and PK are clinical genetic counsellors who were involved in recruitment of patients. SEK was involved in tissue banking and statistical analysis. KAB contributed to molecular analysis. MS contributed to design and validation of BRCA1 and BRCA2 primers for dHPLC mutation analysis. PTS and YK contributed to the MIP copy number assay experiments. DMM obtained consent for tissue banking and surgically collected tumor samples. DH was involved in BRCA1/2 germline mutation analysis and drafting of the manuscript. MF participated in the MIP copy number assay experiments. CBG was involved in study concept and design, pathological analysis, data analysis and interpretation, and drafting of the manuscript. JG participated in whole genome copy number analysis using MIP probes. DGH participated in study concept and design, data analysis and interpretation, and drafting of the manuscript. All authors have read and approved the final version of the manuscript.

## Pre-publication history

The pre-publication history for this paper can be accessed here:


